# Alternative reagents to antibodies in imaging applications

**DOI:** 10.1007/s12551-017-0278-2

**Published:** 2017-07-27

**Authors:** R. Bedford, C. Tiede, R. Hughes, A. Curd, M. J. McPherson, Michelle Peckham, Darren C. Tomlinson

**Affiliations:** School of Molecular and Cellular Biology, Astbury Centre for Structural and Molecular Biology, University of Leeds, Leeds, UK

**Keywords:** Affimer, Adhiron, DARPin, Monobody, Imaging, Super-resolution

## Abstract

Antibodies have been indispensable tools in molecular biology, biochemistry and medical research. However, a number of issues surrounding validation, specificity and batch variation of commercially available antibodies have prompted research groups to develop novel non-antibody binding reagents. The ability to select highly specific monoclonal non-antibody binding proteins without the need for animals, the ease of production and the ability to site-directly label has enabled a wide variety of applications to be tested, including imaging. In this review, we discuss the success of a number of non-antibody reagents in imaging applications, including the recently reported Affimer.

## Introduction

Antibodies raised against a protein of interest have been the main tool used to investigate temporal and spatial protein expression, localisation and behaviour, with immunoglobulin G (IgG) being the most commonly used isotype. In bio-imaging, antibodies have been widely used in a number of techniques, including immunofluorescence microscopy, immunohistochemistry, flow cytometry and immuno-electron microscopy. They are also used in many other research applications, such as immunoprecipitation, enzyme-linked immunosorbent assays and western blotting. The ground-breaking work of Kohler and Milstein in 1975, which resulted in the production of monoclonal antibodies (Kohler and Milstein 1975), has led to the use of these antibodies in treating patients. This began with the licencing of orthoclone OKT3®, a monoclonal antibody for the prevention of tissue rejection in cases of acute kidney transplantation (Starzl and Fung 1986). By 2014, 47 therapeutic-based monoclonal antibody treatments had been approved for use in the USA or Europe, generating almost US $100 billion for the pharmaceutical industry (Ecker et al. 2015).

Despite this success, the generation and validation of antibodies, particularly for research applications, remains challenging, leading to growing concern about the potential for substantial waste of research funds on ‘bad’ antibodies (Taussig et al. 2007; Bordeaux et al. 2010, Bradbury and Pluckthun 2015) and the waste of animals in producing these reagents.

**Fig. 1:**
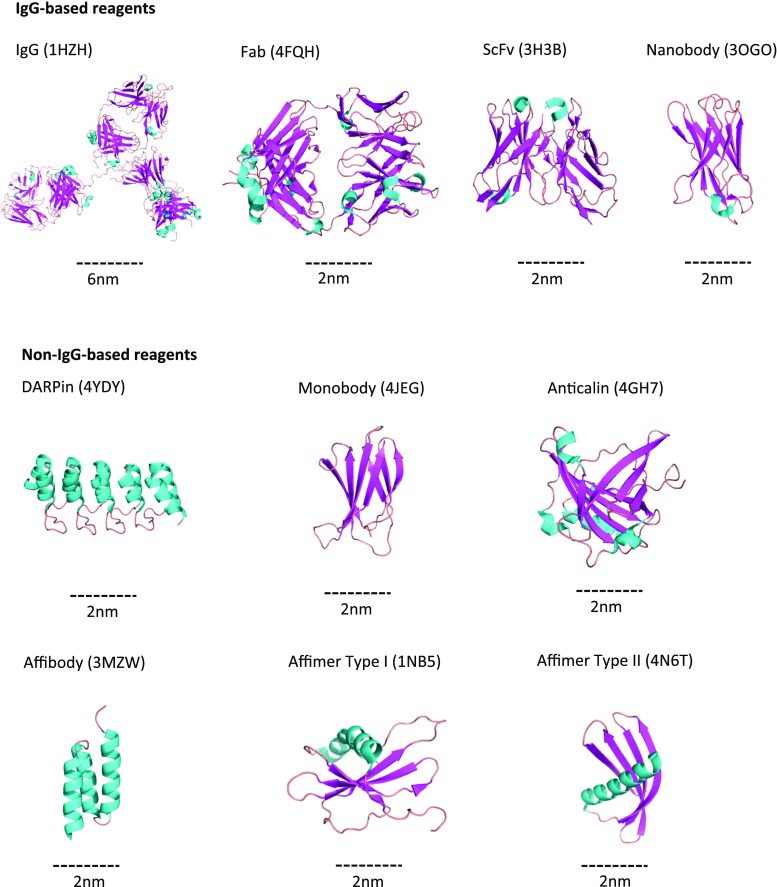
Examples of immunoglobulin G (*IgG*) and non-IgG-based binding reagents developed for use as research tools. One of the major advantages provided by the many IgG alternatives is their smaller size, as demonstrated by the IgG* scale bars* with all alternative reagents compared to scale. IgG-based reagents include the fragmented versions, fragment of antigen binding (*Fab*) and single-chain fragment of variability (*ScFv*) as well as the reformatted Camelid IgG (*Nanobody*). Non-IgG-based reagents [Designed Ankyrin Repeat Proteins (*DARPins*), Monobodies, Anticalins, Affibodies] demonstrate even smaller sizes, with Affibodies approaching 1 nm in diameter compared to the 10-nm diameter measured by the IgG antigen-binding region. PyMOL (Schrödinger, LLC, New York, NY) and KeyNote (Apple Inc., Cupertin, CA) were used to create the images

Recent advances have enabled the production of recombinant antibody fragments in *Escherichia coli* (Fig. 1), allowing a renewable source of reagent and thereby overcoming many issues of batch-to-batch variation commonly observed in animal-produced antibodies. For research purposes, the most commonly used fragments are the fragment of antigen binding (Fab) (Better et al. 1988) and single-chain fragment of variability (ScFv) (Skerra and Pluckthun 1988; Nelson and Reichert 2009). These smaller antibody-derived fragments have the advantages that they can be selected in vitro using a display technology as well as being produced in *E. coli* (Holliger and Hudson 2005; Nelson and Reichert 2009).

More recently, the IgG antibodies from the family*Camelidae* have also been exploited. These antibodies do not contain any light chains, and the heavy chain only contains a single antigen-binding variable domain (V_H_H) (Hamers-Casterman et al. 1993; Muyldermans 2001). The V_H_H, originally referred to as a heavy chain antibody (HCAb), is now known as a single-domain antibody or, more commonly, as a Nanobody (Fig. 1) (Nguyen et al. 2001; Daley et al. 2010). It has been developed for potential therapeutic use by Ablynx (Gent, Belgium). Nanobodies are highly stable, monomeric and smaller than the variable domain (V_H_) of classical antibodies. Importantly, they can be recombinantly produced and purified using *E. coli* to allow large amounts of pure antibody fragment to be generated. Currently, most Nanobodies are still generated using immunisation of *Camelidae*, followed by reformatting of the V_H_H region of the cognate HCAb and further screening by traditional phage display (Nguyen et al. 2001).

As an alternative to antibodies, a number of non-immunoglobulin binding reagents have now been developed, generally through adaptation of a naturally occurring protein or protein domain (Fig. 1). Importantly, all of these reagents are derived from synthetic libraries that allow identification of binding reagents without the use of animals. A potential disadvantage of this approach is that the ability to isolate useful reagents is dependent on the design, size, quality and display format of the library screened. However, important advantages are that these alternative binding reagents can all be produced recombinantly and that they are generally much smaller and more stable than antibodies. Whilst such non-antibody proteins have been reviewed in depth elsewhere (Hey et al. 2005; Skerra 2007; Skrlec et al. 2015; Simeon and Chen 2017), some of the more common reagents will be briefly discussed here (Fig. 1). These include Designed Ankyrin Repeat Proteins (DARPins), Monobodies, Anticalins, Affibodies and more recently Adhirons/Affimers.

DARPins generally contain three to four tightly packed repeats of approximately 33 amino acid residues, with each repeat containing a β-turn and two anti-parallel α-helices (Kawe et al. 2006). This rigid framework provides protein stability whilst enabling the presentation of variable regions, normally comprising six amino acid residues per repeat, for target recognition. Whilst the imaging applications of DARPins will be discussed in detail below, it is worth highlighting the development of a vascular endothelial growth factor (VEGF) A-specific DARPin for the treatment of macular degeneration (Stahl et al. 2013) that has recently entered phase III clinical trials (Molecular Partners AG, Zurich, Switzerland).

Monobodies are based on the fibronectin type III domain (Koide et al. 1998). This domain adopts a β-sandwich structure composed of seven β-sheets and contains three exposed loops available for target recognition. There are two Monobody libraries that diversify amino acids at different positions in the scaffold. Whilst the original libraries diversified amino acids within the loop regions, the second-generation library also diversifies a segment of the β-sheet. (Koide et al. 1998, 2012). This ‘side and loop’ diversification enables presentation of a concave binding surface, as opposed to the more usual flat or convex paratope of Monobodies. These different binding conformations increase the range of targets available for selection with those involved in protein–protein interactions favoured by this ‘side and loop’ library (Wojcik et al. 2016).

Anticalins are derived from the lipocalin scaffold (Vogt and Skerra 2004). They contain eight anti-parallel β-strands that form a conserved β-barrel, attached to an adjacent α-helix. The β-barrel provides target recognition by supporting four solvent-exposed loops (Gebauer and Skerra 2012). These reagents are derived from the human lipocalin protein family and as a consequence have low immunogenicity [Pieris AG (Freising, Germany) and AlgoNomics NV (Gent, Belgium), 2006]. An anti-hepcidin Anticalin is already in phase I clinical trials for the treatment of anaemia (Moebius et al. 2015).

Affibodies, based on the B-domain of staphylococcal protein A, adopt a folded α-helical structure that provides Affibodies with their stability (Nord et al. 1997). Further efforts to improve the innate stability of the B-domain led to a mutated ‘Z-domain’, with the ability of Affibodies to recognise a variety of targets through the randomisation of amino acid residues in the first two helices. This is the region of the protein that binds the Fc region of IgG in protein A (Lofblom et al. 2010).

Affimers can be classified as type I and type II based on their scaffold of either the human stefin A protein (Hoffmann et al. 2010) or consensus plant phytocystatin protein, respectively (Tiede et al. 2014). Both types contain four β-sheets and an α-helix. The binding region is generated from sequences in two variable loops presented between pairs of β-sheets. Affimers have been raised against a diverse set of targets, thereby demonstrating their utility in many different molecular biology applications, including those related to bio-imaging (Fisher et al. 2015; Kyle et al. 2015; Raina et al. 2015; Sharma et al. 2016; Arrata et al. 2017; Koutsoumpeli et al. 2017; Tiede et al. 2017; Wang et al. 2017b).

Whilst non-antibody binding proteins were originally generated as simple affinity reagents, their ability to bind functional surfaces of target proteins has fast-tracked their use as therapeutic reagents (Roovers et al. 2007; Tamaskovic et al. 2012; Sha et al. 2017). Their small size (Fig. 1), specificity and stability have also proven important in exploiting them as imaging tools, particularly for use in ‘super-resolution’ imaging. The bio-imaging applications of these reagents are described in more detail below.

## Binding reagents for use as imaging tools

### Detecting cancer biomarkers using antibody alternatives

Although a large repertoire of antibodies are available for the detection of cancer biomarkers in tissues (Bouchelouche et al. 2010; Wang et al. 2013; Howat et al. 2014), the beneficial properties of alternative non-antibody reagents, particularly their specificity and ease of production, has prompted a number of groups to investigate their use in this application (Orlova et al. 2007; Goldstein et al. 2015; Van Audenhove and Gettemans 2016).

The human epidermal growth factor receptor 2 oncogene (HER-2) drives a number of oncogenic processes, including proliferation and invasion (Slamon et al. 1987). Clinically, the detection of HER-2 not only provides a prognostic prediction but also guides therapeutic options with Trastuzumab, a monoclonal antibody able to treat HER-2 over-expressing cancer cells (Vogel et al. 2002; Seidman et al. 2008). The importance of detecting HER-2 in tissue biopsies has prompted novel methods to be developed to detect this biomarker. A highly specific DARPin has been isolated for use in immunohistochemical applications for the detection of HER-2, with results proving to be as reliable, but with improved specificity, over a U.S. Federal Food and Drug Administration (FDA)-approved antibody (4B5) (van der Vegt et al. 2009) for the detection of HER-2 in human tissues (Theurillat et al. 2010). An anti-HER-2 Affibody is also currently being commercially developed by Abcam (Cambridge, UK) for use in immunohistochemistry. It is also worth noting that HER-2-specific Nanobody reagents have been developed for use in tissue staining, although these are mainly used as molecular imaging tools in vivo and will be discussed in the next section of this review (Vaneycken et al. 2011; Xavier et al. 2013).

Nanobodies have, however, been raised against a number of other cancer biomarkers for use in tissue staining. A nanobody targeting the antigen ADP-ribosyltransferase ARTC2.2 has been used for the histological staining of ARTC2-positive xenografts for the validation of in vivo imaging results post-sacrifice of the test rodent (Bannas et al. 2015). The results demonstrated that in comparison to an anti-ARTC2 antibody, a strong and homogenous staining of cells was observed in positive tumour sections in comparison to a much weaker and non-homogenous staining pattern shown by the antibody. This is likely a consequence of the larger antibody being less efficient at tumour penetration (Bannas et al. 2015).

Affimers have recently been used to target VEGF receptor 2 (VEGFR2), a key protein in blood vessel formation in tumours (Tiede et al. 2017). In histochemical staining, Affimers showed a similar staining pattern to but greater sensitivity than a commercially available anti-VEGFR2 antibody. Again, one proposal for this enhanced sensitivity is an improved ability to penetrate tissues due to the smaller size of the Affimer. In the same report, Affimers that target Tenascin C (TNC) were also shown to be effective in histochemistry techniques. Staining patterns were similar to those observed by an anti-TNC antibody, albeit with slightly reduced sensitivity in this case, but again demonstrating the utility of Affimers as reagents for the detection of tumour biomarkers in tissue (Tiede et al. 2017).

Of course, to make these types of reagents more useful to the wider scientific community, at least at this stage of their development, it might be appropriate to generate them as Fc fusions, thereby directly replacing antibody binding without the need to change current detection protocols.

### Alternative binding reagents for tumour imaging in vivo

Although the detection of cancer biomarkers in tissue samples is useful for predicting prognosis and identifying the treatment path, the ability to image tumours in vivo and non-invasively is emerging as an important tool in cancer diagnosis (Stern et al. 2013; Vazquez-Lombardi et al. 2015). The smaller size and lack of Fc region of these alternative reagents enables much greater tumour penetration whilst also allowing rapid clearance from surrounding tissues, thus enhancing both the sensitivity and specificity of visualisation (Cuesta et al. 2009; Stern et al. 2013; Luo et al. 2015).

The diagnostic strategy currently employed for the detection of prostate cancer suffers from a low cancer detection rate and, consequently, more specific detection tools are required (Babaian et al. 2000). A number of antibody alternative reagents targeting prostate-specific membrane antigen (PSMA), a prostate cancer biomarker, have been developed (Chatalic et al. 2015; Han et al. 2016, Mazzocco et al. 2016). Their ability to recognise an extracellular epitope on PSMA has enabled the development of tests for use against viable tissues. This represents a marked improvement on currently approved monoclonal antibodies such as ProstaScint, which recognises an intracellular epitope on PSMA, thus limiting staining to necrotic tissues (Bander 2006; Chatalic et al. 2015; Barinka et al. 2016). Using an ScFv to target PSMA has improved diagnostic capabilities through reduced background labelling of endogenous Fc receptors in surrounding tissues (He et al. 2010; Mazzocco et al. 2016). Nanobodies and anticalins have shown similar promise in targeting PSMA, displaying good tumour targeting and rapid blood clearance (Chatalic et al. 2015; Barinka et al. 2016).

As discussed above, HER-2 has been targeted by a number of binding proteins. The success of antibody alternatives as HER-2 imaging tools in vivo has been highlighted by studies conducted using DARPins and Nanobodies. Increased sensitivity and lower background has been observed when imaging HER-2 and epidermal growth factor receptor (EGFR) biomarkers (Mortimer et al. 2014). The reduced off-target effects of Nanobodies are evidenced by their much faster clearance rates from non-specific tissues compared to monoclonal antibodies. The radiolabelled HER-2-targeting monoclonal antibody Trastuzumab (Delaney 1999) and the EGFR-targeting antibody Cetuximab (Prewett et al. 1996), both clinically approved, clear very slowly from non-specific tissues (>24 h). By comparison, the anti-EGFR Nanobody clears within 45 min of administration (Kruwel et al. 2016). Affimers have also been demonstrated for use as ex vivo imaging tools for the detection of tumour biomarkers (Tiede et al. 2017). The reduced circulation time and more rapid tumour penetration afforded by smaller binding proteins compared to antibodies provides the potential for a faster and timelier imaging procedure and thus should reduce patient time in hospital. Another approach with promise for in vivo tumour imaging involves dye-conjugated Affimers that have been used in Förster resonance transfer (FRET) experiments (Conway et al. 2014; Wang et al. 2017b).

The ability to use alternative-binding proteins to detect cancer biomarkers, as outlined in Table 1, has led researchers to attempt to develop their therapeutic potential by combining tumour detection with treatment, a field termed *theranostics*. Radionuclide-labelled antibodies have previously been explored for use in radio immunotherapy (RIT), a technique that exploits a tumour-targeting molecule to act as a vehicle for the transport of cytotoxic compounds to the tumour. Thirteen monoclonal antibodies are currently approved by the FDA for use in RIT, with the majority targeting blood-borne carcinomas (Reichert 2014; Ecker et al. 2015). Monoclonal antibodies that target cancers originating from epithelial tissues have so far been less successful (Weiner and Adams 2000) because of their slow diffusion rate to their target site due to their size. The improved tumour penetration and faster tissue clearance linked to smaller binding reagents has led to a number of efforts to use them in RIT, alternatively called targeted radionuclide therapy (TRNT). The use of Nanobodies in TRNT has been shown for the combined detection and therapy of human growth factor-expressing cancers (Vosjan et al. 2012). Additionally, the Nanobody previously described for the detection of HER-2 has been conjugated to the radio lanthanide, lutetium-177, for this purpose (D'Huyvetter et al. 2014). Affibodies are another reagent class used for TRNT for the treatment of HER-2 disposed cancers, with lutetium-177-tagged anti-HER-2 Affibodies demonstrating remarkable inhibition of tumour formation (Tolmachev et al. 2007).

**Table 1:** Antibody alternatives for use as in vivo tumour imaging tools

Reagent	Target^a^	Tumour-association	Reference
Nanobody			
8B6, 7C12, 7D12	EGFR	Epidermoid and prostate	Huang et al. 2008; Gainkam et al. 2011; Oliveira et al. 2012; Kruwel et al. 2016
2Rs15d, 11A4	HER-2	Colon, breast and ovarian	Vaneycken et al. 2011; Xavier et al. 2013
1E2, 6E10	HGF	Glioblastoma	Vosjan et al. 2012
α-MMR	MMR	Mammary adenocarcinoma, Lewis lung carcinoma	Movahedi et al. 2012
CEA1	CEA	Colon	Vaneycken et al. 2010
JVZ-007	PSMA	Prostate	Chatalic et al. 2015
DARPin			
(HE)3-G3	HER-2	Colon, breast and ovarian	Mironova et al. 2014; Goldstein et al. 2015
Monobody			
E1	hEphA2	Lung, breast and colon	Park et al. 2015
Anticalin			
PRS-110	HGFR	Various	Terwisscha van Scheltinga et al. 2014
PRS-050	VEGF-A	Various	Meier et al. 2014
A3	PSMA	Prostate	Barinka et al. 2016
Affibody			
ABY-025	HER-2	Colon, breast and ovarian	Sandberg et al. 2017
affiFAP	EGFR	Epidermoid and prostate	Wang et al. 2017a
ZHPV16E7384	HPV16 E6/E7	Cervical	Xue et al. 2016
	CAIX	Renal	Garousi et al. 2016
99mTc-ZIGFR:4551-GGGC	IGF-1R	Various	Mitran et al. 2015
HEHEHE-z08698-NOTA	PDGFRβ	Various	Rosestedt et al. 2015
Affimer			
Anti-TNC Affimer	TNC	Various	Tiede et al. 2017

^a^ *EGFR* epidermal growth factor receptor, *HER-2* human epidermal growth factor receptor-2,* HGF* hepatocyte growth factor, *MMR* macrophage mannose receptor, *CEA* carcinoembryonic antigen, *PSMA* prostate specific membrane antigen, *hEphA2* human eryhoropoietin-producing hepatocellular A2, *HGFR* hepatocyte growth factor receptor, *VEGF-A* vascular endothelial growth factor A, *HPV16 E6/E7* human papillomavirus 16 E6 and E7 oncoproteins, *CAIX* carbonic anhydrase 9, *IGF-1R* insulin-like growth factor 1 receptor, *PDGFRβ* platelet-derived growth factor beta, *TNC* tenascin C

Despite promising preliminary data for the use of antibody alternatives in TRNT, issues surrounding renal accumulation of radioactively labelled proteins requires further study (Gainkam et al. 2011; Vosjan et al. 2012). In the meantime, one possible approach is their use in pre-targeting, i.e. the targeting of pre-tagged binding reagents to a tumour location prior to the administration of radionuclides able to recognise the tag (Honarvar et al. 2016). An alternative proposal is the fusion of binding reagents to larger proteins to extend half-life; however this approach may result in further problems by causing the toxic compounds to accumulate in different tissues (Vosjan et al. 2012).

### Use of antibody alternatives as fluorescent imaging probes

Fluorescent labelling of antibodies is a common approach for the detection and localisation of proteins in fixed cells. A major advantage of non-antibody binding proteins is their ability to be engineered at specific sites for site-directed modifications. In this context, the addition of a single cysteine residue to multiple non-antibody binding proteins, including Affimers, has allowed the site-specific addition of a fluorophore and alternative labels (Fisher et al. 2015; Tiede et al. 2017). Fluorescently labelled Affimers have been used to detect an antigen of herpesvirus of turkeys in infected cells lines, as well as in cell imaging by the fluorescent detection of TRPV1, a ligand-activated non-selective calcium-permeant cation channel (Tiede et al. 2017). In this study, live cells were incubated with an Affimer, and post-fixation localisation of the Affimer was detected using an antibody to a fusion tag; in addition co-localisation was observed with an anti-TRPV1 antibody. A further useful property of these reagents is their ability to function in the cytosol, thereby enabling live cell imaging. Nanobodies have exploited this property to visualise cytokeratin-B and lamin Dm0 in their dynamic states (Rothbauer et al. 2006), and a Nanobody raised against a HIV-1 precursor protein has enabled the observation of viral particle assembly in real-time (Helma et al. 2012).

In conventional fluorescence microscopy, spatial resolution is limited by the wavelength of light to approximately 200 nm at best, as discovered by Abbe (Abbe 1873). In the last few years, various approaches have been developed to overcome this limit, collectively termed as ‘super-resolution’ light microscopy (Evanko 2009; Patterson 2009; Galbraith and Galbraith 2011). In particular, single molecule localisation microscopy (SMLM) techniques, such as PALM (photoactivated localisation microscopy), STORM (stochastic optical reconstruction microscopy) and dSTORM (direct STORM), can localise the positions of single fluorophores with very high accuracy, providing resolutions of approximately 20 nm. PALM uses genetically encoded fluorescent proteins (Betzig et al. 2006), while STORM (Rust et al. 2006) and dSTORM (Heilemann et al. 2008) use fluorescently labelled antibodies.

The best localisation precision tends to be obtained using synthetic dyes rather than fluorescent proteins because dyes emit higher numbers of photons and the localisation precision depends on the number of photons collected (Moerner 2012). However, this approach is limited by the fact that the dyes are conjugated to antibodies and thus are placed some distance away from the target protein. This means that the localisation accuracy is limited by the size of the antibody. In traditional immunofluorescence techniques, in which both a target-specific primary and a secondary detection antibody are used, the dye can be placed as far away as approximately 30 nm from the intended target. This distance is referred to as ‘linkage error’. Even directly conjugating the dye to a primary antibody (150 kDa; approx. 10 nm in size) still results in a linkage error of approximately 10 nm (Ries et al. 2012), and there may be multiple fluorophores per antibody. Whilst not important for most diffraction-limited immunofluorescence experiments, in super-resolution approaches this limitation significantly reduces the potential resolving power. The small size of alternative binding reagents reduces this linkage error, making these reagents particularly attractive for use in super-resolution microscopy.

SMLM approaches have begun to exploit the small size of Nanobodies, for example the anti-green fluorescent protein (GFP) Nanobody (13 kDa, approx. 2 nm in size) used to target GFP–fusion proteins reduced the linkage error to approximatley 2 nm (Ries et al. 2012). Similar results have been obtained using Nanobodies against nuclear pore complex proteins (Pleiner et al. 2015). Nanobodies have also been used in DNA-PAINT (DNA-points accumulation in nanoscale topography) (Jungmann et al. 2010, 2014; Agasti et al. 2017). The anti-HER-2 Affibody has also shown promise in super-resolution microscopy, enabling visualisation of the intra- and intercellular distribution patterns of HER-2 in over-expressing cancer cells (Peckys et al. 2015).

The small size of Affimers (approx. 10–12 kDa, approx. 2 nm), similar to that of Nanobodies, and the ability to specifically label certain sites makes them useful tools in super-resolution microscopy. Affimers have recently been obtained that specifically bind to tubulin and have been used in dSTORM [with both total internal reflection fluorescence (TIRF) and three-dimensional techniques]. Interestingly, the Affimer labels interphase microtubules in a similar way to a widely used antibody. In addition, the Affimer labels the central region of the cytokinetic furrow, a region from which antibodies are usually excluded owing to the density of tubulin in this region, highlighting a further advantage of using smaller probes. HER-4 Affimers have also demonstrated their value in single molecule tracking (Tiede et al. 2017).

## Conclusion

After many years of antibodies dominating molecular recognition techniques in biology, the field now has the ability to use alternatives to move away from the reliance on animal-produced reagents. Over 50 non-antibody scaffolds with target recognition capabilities have been developed that provide a number of advantages over traditional antibodies. In addition to being produced without the use of animals, these non-antibody scaffolds demonstrate a higher stability and can be produced as recombinant proteins in *E. coli*. Their smaller size is particularly useful for imaging targets for two reasons. First, they penetrate tissues and can access epitopes in densely packed subcellular structures of cells more readily than antibodies, an advantage for both imaging tumours and in ‘super-resolution’ microscopy. Second, they place the fluorophore closer to the target of interest, providing an increased spatial resolution in ‘super-resolution’ approaches. We anticipate that such antibody alternatives will become widely used in a range of biological and medical imaging applications.
